# Gene knockout of nuclear progesterone receptor provides insights into the regulation of ovulation by LH signaling in zebrafish

**DOI:** 10.1038/srep28545

**Published:** 2016-06-23

**Authors:** Haipei Tang, Yun Liu, Jianzhen Li, Yike Yin, Gaofei Li, Yu Chen, Shuisheng Li, Yong Zhang, Haoran Lin, Xiaochun Liu, Christopher H. K. Cheng

**Affiliations:** 1 State Key Laboratory of Biocontrol, Institute of Aquatic Economic Animals and Guangdong Province Key Laboratory for Aquatic Economic Animals, School of Life Sciences, Sun Yat-Sen University, Guangzhou 510275, China; 2 School of Biomedical Sciences, The Chinese University of Hong Kong, Hong Kong, China; 3 School of Biomedical Sciences Core Laboratory, The Chinese University of Hong Kong Shenzhen Research Institute, Shenzhen 518057, China

## Abstract

It is well established that the luteinizing hormone surge triggers ovulation, a dynamic process leading to the release of the mature oocyte from the ovarian follicle. But how this process controlled by LH signaling remains largely unknown in non-mammalian species. In this study, we investigated the roles of nuclear progesterone receptor (*npr*) in LH-induced ovulation. Our results indicate that the nuclear progesterone receptor serves as an important mediator of LH action on ovulation. This conclusion is based on the following results: (1) the expression level of *npr* peaks at the full-grown stage of the follicles; (2) the expression of *npr* is stimulated by LH signaling *in vitro* and *in vivo*; and (3) the *npr* null females are infertile due to ovulation defects. Moreover, we further show that LH signaling could induce *ptger4b* expression in an *npr*-dependent manner, and blockage of Ptger4b could also block hCG-induced ovulation. Collectively, our results not only demonstrate that *npr* serves an indispensable role in mediating the action of LH on ovulation in zebrafish, but also provide insights into the molecular mechanisms of the regulation of ovulation in fish.

Luteinizing hormone (LH) signaling has attracted much research attention for a long time because of its critical roles in reproduction^1^,^2^,^3^,^4^,^5^. In mammals, LH signaling has been extensively studied using gene knockout mice^1^,^3^,^6^,^7^. Deletion of *LHβ* causes infertility in both males and females in mice^3^. Recently, we have established the *lhβ* knockout model in zebrafish and found that final oocyte maturation and ovulation were disrupted in the mutants^2^. Interestingly, we also found that the gonad-specific *igf3* serves as an important mediator of the action of LH on oocyte maturation^8^. However, the downstream factors in mediating the action of LH on ovulation are largely undetermined.

The nuclear progesterone receptor (*nPR;* official symbol *PGR*), which is a member of the nuclear receptor transcription factor superfamily, has been proposed as an important factor for LH-dependent ovulation in mammals. Studies in mice and in human have demonstrated the critical role of *nPR* in ovulation^9^,^10^. Female mice lacking *nPR* exhibit anovulation, impaired sexual behavior, uterine dysfunction, impaired ductal branching morphogenesis and lobuloalveolar differentiation of the mammary gland^11^,^12^,^13^,^14^. Although the functional roles of *nPR* in the ovulation of rodents and human are well recognized^15^,^16^, its functional roles in non-mammalian species are not well understood.

Ovulation is a precise and complex biological process during which one or more mature fertilizable oocytes are released from the surrounding follicle wall into the ovarian cavity^17^. The molecular mechanisms for ovulation have been investigated in many studies. Previous studies have shown that control of the ovulation process may involve the cooperative action of a number of regulators including a variety of proteases^18^,^19^,^20^, matrix metalloproteinases (*mmps*)^21^,^22^,^23^ and tissue inhibitors of metalloproteinases (*timps*)^22^,^23^,^24^, *etc*. Furthermore, prostaglandins (PGs) also participate in regulating key aspects of the ovulatory process^25^,^26^. However, how *npr* and these factors act to mediate the stimulatory action of LH on ovulation has yet to be established.

In this study we have investigated whether *npr* could mediate the action of LH on ovulation in zebrafish. Our data indicated that *npr* is stimulated by LH signaling *in vitro* and *in vivo*. Moreover, the *npr* null fish are infertile due to ovulation defects. Further studies suggest that *ptger4b* is a downstream factor of *npr* in mediating the action of LH on ovulation. These results revealed that *npr* and its downstream factor *ptger4b* are indispensable for ovulation in zebrafish.

## Results

### Differential gene expression of *mprα*, *mprβ an*d *npr* during zebrafish folliculogenesis

**Figure 1:**
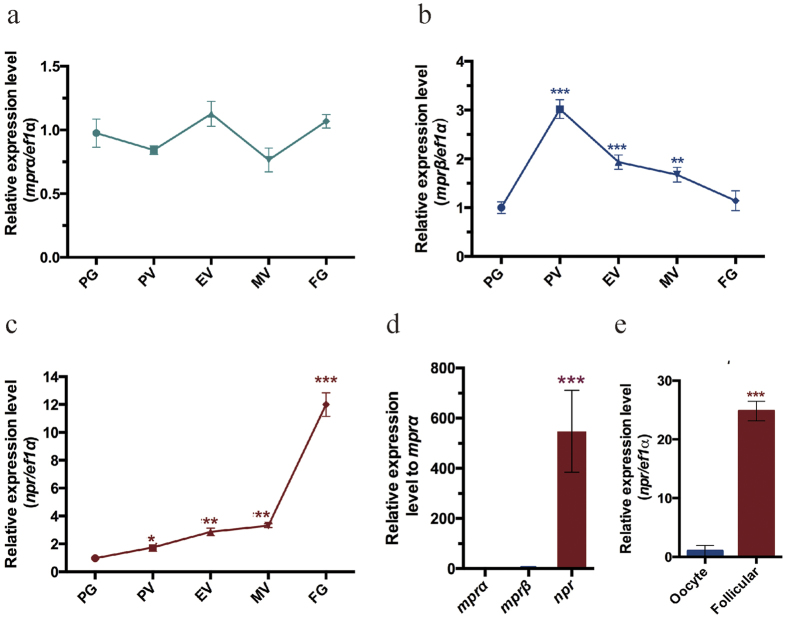
Expression of *mprα, mprβ* and *npr* during folliculogenesis. (**a–c**) Real-time PCR detection the expression of *mprα* (**a**), *mprβ* (**b**) and *npr* (**c**) in the follicles of different stages isolated from the ovaries of adult zebrafish. (**d**) The relative expression of *mprα*, *mprβ* and *npr* in FG stage follicles isolated from the ovaries of adult zebrafish. (**e**) The expression of *npr* in the oocyte and follicular cells isolated from FG stage follicles. Each value represents the mean value ± SEM of quadruplicate assays of 4 independent experiments (*P < 0.05; **P < 0.01; ***P < 0.001 vs control).

Three progesterone receptors including the nuclear progestin receptor (*npr*) and membrane progestin receptors (*mprα* and *mprβ*) have been identified in zebrafish. We first examined the relative levels of the three progesterone receptors in the ovarian follicles of different developmental stages using real-time PCR. The expression of *mprα* was not significantly changed during zebrafish folliculogenesis (Fig. 1a). However, the level of *mprβ* increased from primary growth (PG) stage and reaching the highest level in previtellogenic (PV) stage, and then decreased afterwards (Fig. 1b). The expression of *npr* gradually increased with a sharp increase from the midvitellogenic (MV) stage to full grown but immature (FG) stage (Fig. 1c). At the FG stage, *npr* was the most abundantly expressed progesterone receptor (Fig. 1d). The expression of *npr* was significantly higher in the follicular cells than the oocyte (Fig. 1e). These results suggest that *npr* may play an important role in final oocyte maturation or in the ovulation of zebrafish.

### Regulation of *npr* expression by LH signaling *in vitro* and *in vivo*

**Figure 2:**
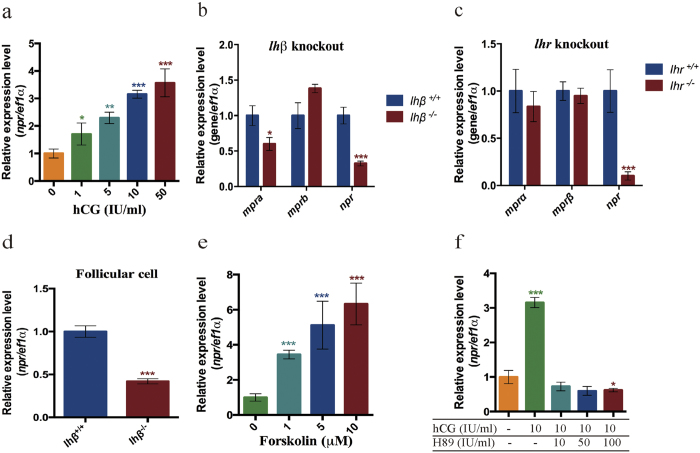
Regulation of *npr* expression by LH signaling. (**a**) Dose dependence of hCG treatment for 2 hours on the expression of *npr* in the intact FG stage follicles, indicating that the expression of *npr* is regulated by LH signaling *in vitro*. (**b,c**) Relative expression of *mprα*, *mprβ* and *npr* in intact FG stage follicles of the *lhβ* mutant and *lhr* mutant, indicating that the expression of *npr* is regulated by LH signaling *in vivo*. (**d**) Relative expression of *npr* in denuded follicular cells isolated from FG stage follicles of the wild-type fish and *lhβ* mutant, demonstrating that the regulation of *npr* expression was occurrs at the follicular cells. (**e**) Dose response of forskolin treatment for 2 hours on the expression of *npr* in the primary cultured follicular cells. (**f**) Treatment by hCG on the expression of *npr* in the presence or absence of H89 in the primary cultured follicular cells, demonstrating that the cAMP-PKA pathway is involved in the regulation of *npr* expression by LH signaling *in vitro*. Each value represents the mean value ± SEM of quadruplicate assays of 3 independent experiments (*P < 0.05; **P < 0.01; ***P < 0.001 vs control).

We then examined whether the expression of *npr* is regulated by LH signaling. FG stage follicles were incubated with different concentrations of human chorionic gonadotropin (hCG). After two hours of incubation, the *npr* expression was dramatically increased in a dose dependent manner (Fig. 2a). Moreover, the *npr* expression was significantly decreased in the *lhβ* or *lhr* mutants (Fig. 2b–d), indicating that *npr* expression is also regulated by endogenous LH signaling. However, the *mprα* expression was only significantly decreased in the *lhβ* mutant but not the *lhr* mutant, and *mprβ* mRNA was not significantly changed in the *lhβ* or *lhr* mutant (Fig. 2b,c). These data indicate that the expression of *npr* is regulated by LH signaling *in vitro* and *in vivo*.

LH signaling mainly acts via the cAMP-PKA pathway in zebrafish^27^. We therefore investigated whether this pathway is indeed involved in the regulation of *npr* expression. Two pharmalogical agents, forskolin (an activator of adenylate cyclase to increase the intracellular cAMP level) and H89 (an inhibitor of PKA to decrease the intracellular cAMP level) were used in the experiments. Treatment of cultured zebrafish follicular cells with forskolin for 2 h significantly increased *npr* expression (Fig. 2e), and H89 effectively suppressed hCG (50 IU/ml) induced *npr* expression (Fig. 2f). These data demonstrate that the expression of *npr* is regulated by LH signaling through the cAMP-PKA pathway.

### Gene fragment deletion of *npr* in zebrafish

**Figure 3:**
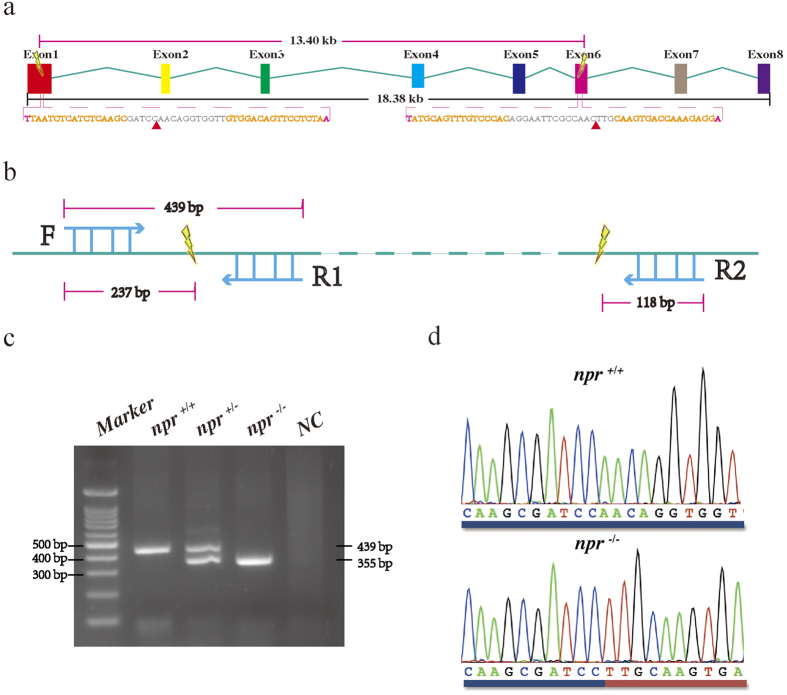
Targeted fragment deletion of the zebrafish *npr* gene. (**a**) Location of the TALEN binding sites on the zebrafish *npr* gene. Two TALEN target sites in exon 1 and exon 6 were designed to delete the 13.4 kb flanking region. The mutated genotype missing the genomic sequences between the two red triangles was analyzed in this study. (**b**) The primer design strategy to detect *npr* fragment deletion in this study. (**c**) A representative agarose gel analysis of PCR genotyping. Three primers shown in (**b**) were used in the genomic PCR to distinguish the *npr*^+/+^, *npr*^+/*−*^ and *npr*^−/−^ genotypes. (**d**) DNA sequence analysis of the *npr*^−/−^ with large genomic deletion.

To assess the *in vivo* function of *npr* in mediating LH signaling in fish, we have established an *npr* gene knockout line using TALENs. There are eight exons in the zebrafish *npr.* Two TALEN target sites were designed in the first and the sixth exon to delete the flanked genomic fragment (13.40 kb) (Fig. 3a). The *npr* fragment deletion could be ascertained by properly designed primers (Fig. 3b). Successful *npr* fragment deletion was confirmed by genomic PCR (Fig. 3c) and sequencing (Fig. 3d).

### Ovulation is disrupted in the *npr* mutant

**Figure 4:**
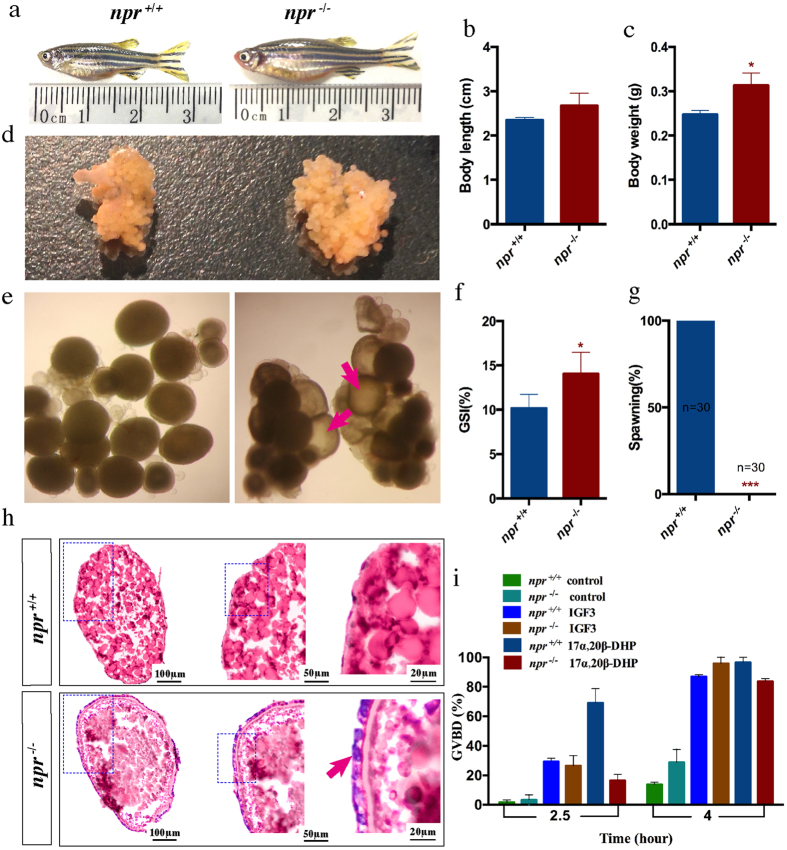
Phenotype analysis of the *npr*^+/+^ and *npr*^−/−^ female zebrafish at 75 dpf. (**a**) Gross morphology of the *npr*^+/+^ and *npr*^−/−^ fish. (**b,c**) Body length (**b**) and body weight (**c**) of *npr*^+/+^ and *npr*^−/−^ mutants (n = 6). (**d**) Ovary morphology of the *npr*^+/+^ and *npr*^−/−^ fish. (**e**), Microscopic examination of follicles from *npr*^+/+^ and *npr*^−/−^ mutants. Follicles were collected at 15:00 pm and matured oocytes (red arrows) could be observed in the *npr*^−/−^ mutant. (**f**) The gonado somatic index (GSI) of *npr*^+*/*+^ and *npr*^−/−^ fish (n = 6). (**g**) Spawning assay. No *npr*^−/−^ fish succeeded in spawning (n = 30). (**h**) Ovary histology of the *npr*^+*/*+^ and *npr*^−/−^ fish. The outer layer cells of the matured follicles (indicated by arrow) failed to breakdown in the *npr*^−/−^ fish. (**i**) Oocyte maturation assay. FG stage follicles from *npr*^+*/*+^ and *npr*^−/−^ fish were treated with IGF3 or 17α, 20β-DHP, and the percentages of follicles that underwent germinal vesicle breakdown (GVBD) were recorded. Each value represents the mean value ± SEM (n = 4) of three independent experiments (*P < 0.05; ***P < 0.001 vs *npr*^+/+^).

The *npr* mutants were viable and developed normally. The genotypes were inherited in the expected Mendelian ratio. Interestingly, only female mutants were infertile while males were fertile. The body size and the abdomen of the female *npr* mutants were bigger compared with the wild-type at adult stage (Fig. 4a). The body length was not affected but the body weight was significant higher in the mutants (Fig. 4b,c). A significant increase in the ovary size and the gonadosomatic index (GSI) was found in the *npr* mutants (Fig. 4d,f). Further examination of the ovaries showed that mature follicles were present in the mutants but not in the wild-type fish at 15:00 in the afternoon (Fig. 4e). Successful spawning in the morning was observed in the wild-type fish but not in the mutants (Fig. 4g). Ovarian histology analysis showed that follicles of different developmental stages could be found in the ovary of the *npr* mutants (Fig. 4h). The mature oocytes were trapped within the follicular cells in the mutants, indicating that the *npr* null fish fail to ovulate (Fig. 4h). Treatment of FG stage follicles from the *npr* mutant with 17α, 20β-DHP or IGF3 could induce oocyte maturation, suggesting that these FG stage follicles from the mutant fish are of good quality (Fig. 4i). However, injection of hCG could not rescue the ovulatory defect of the *npr* null mutants, suggesting that *npr* acts as an downstream factor of LH signaling. These data indicate that disruption of *npr* mainly causes ovulation defects in zebrafish.

### *Ptger4b* is regulated by LH signaling in an *npr*-dependent manner

**Figure 5:**
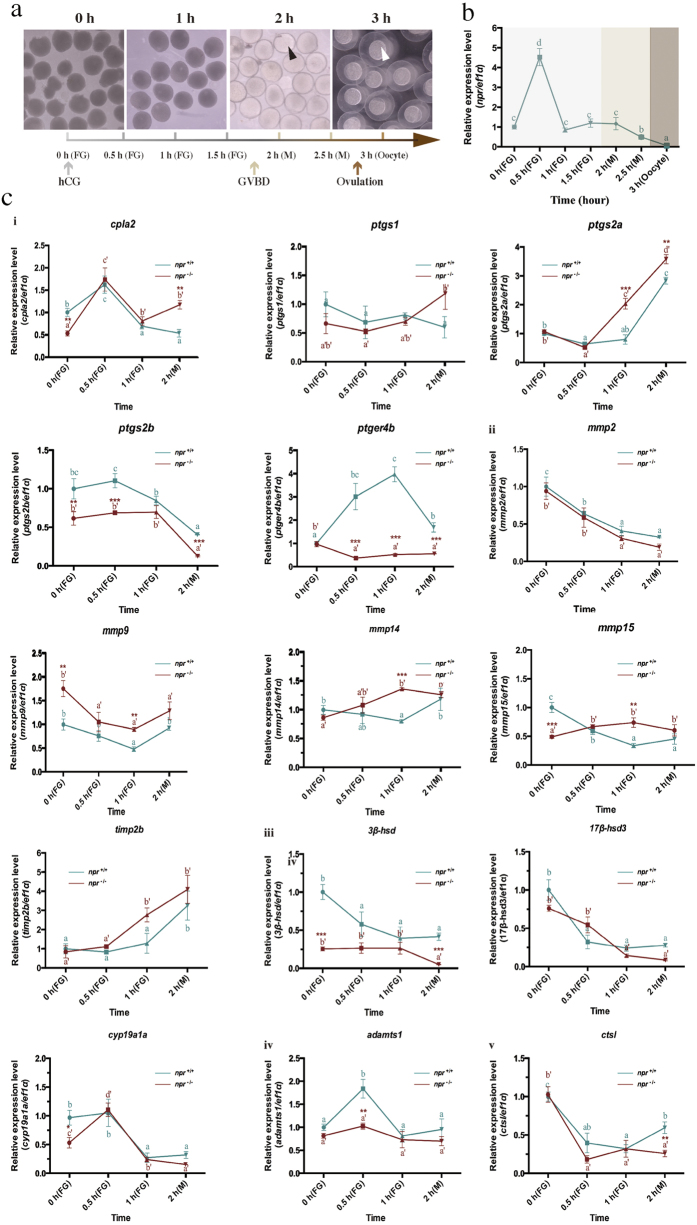
Regulation of ovulation-related genes by LH signaling in *npr*^+/+^ and *npr*^−/−^ fish. (**a**) The timescale of hCG-induced oocyte maturation and ovulation in *npr*^+/+^ zebrafish. Follicles were isolated at different time points after hCG (10 IU/μl) injection. Matured follicle with transparent appearance is indicated by black arrow and ovulated oocyte with enlarged fertilization membrane is indicated by white arrow. (**b**) The expression profile of *npr* after hCG injection. (**c**) The expression profile of ovulation related genes in *npr*^+/+^ and *npr*^−/−^ fish after hCG injection. (i) Genes involved in prostaglandin biosynthesis; *mmps* and tissue inhibitors of metalloproteinases (*timp2b*); (iii) Genes involved in steroidogenesis; (iv) A disintegrin and metalloprotease with thrombospondin motifs1 (*adamts1*) and (v) cathepsin L (*ctsl*). The expression levels were normalized to that of *ef1α* and then to the expression level in *npr*^+/+^ fish at 0 h. Different letters in each dataset indicated statistical significance (*P* < 0.05). Each value represents the mean value ± SEM (n = 4) of three independent experiments (*P < 0.05; **P < 0.01; ***P < 0.001 vs *npr*^+/+^).

To further analyze the molecular mechanism underlining anovulation observed in the *npr* mutants, we have established an *in vivo* ovulation system in zebrafish by intraperitoneal injection of hCG (10 IU/μl) to mimic the endogenous LH surge to induce oocyte maturation and ovulation. The timescale of the hCG-induced oocyte maturation and ovulation in zebrafish is shown in Fig. 5a. Maturation of the follicles and ovulation of the oocytes were observed around 2 hours and 3 hours after hCG injection respectively (Fig. 5a). The temporal expression profiles of *npr* after hCG injection were analyzed. The expression of *npr* was drastically up-regulated at 0.5 hour and then decreased afterwards (Fig. 5b), further confirming that *npr* expression is regulated by LH signaling.

The ovulation-related genes could be regulated by LH signaling in an *npr*-dependent or *npr*-independent manner. The expression profiles of the ovulation-related genes, including the prostaglandin biosynthesis genes, matrix metalloproteinases *(mmps)* and tissue inhibitors of *mmps*, steroidogenesis genes, *adamts1* and *ctsl* were analyzed in the wild-type and *npr*^−/−^ mutant after hCG injection (Fig. 5c). *Ptger4b* and *adamts1* expression was significantly up-regulated in the *npr*^+/+^ but not in the *npr*^−/−^ fish after hCG injection, indicating that these genes were induced by LH signaling through activation of *npr*. Other genes including *cpla2*, *ptgs2a* and *timp2b* were up-regulated in both *npr*^+/+^ and *npr*^−/−^ fish after hCG injection, suggesting that these genes may be induced by LH signaling in an *npr*-independent manner.

### *Ptger4b* is a downstream mediating factor of *npr* in ovulation

**Figure 6:**
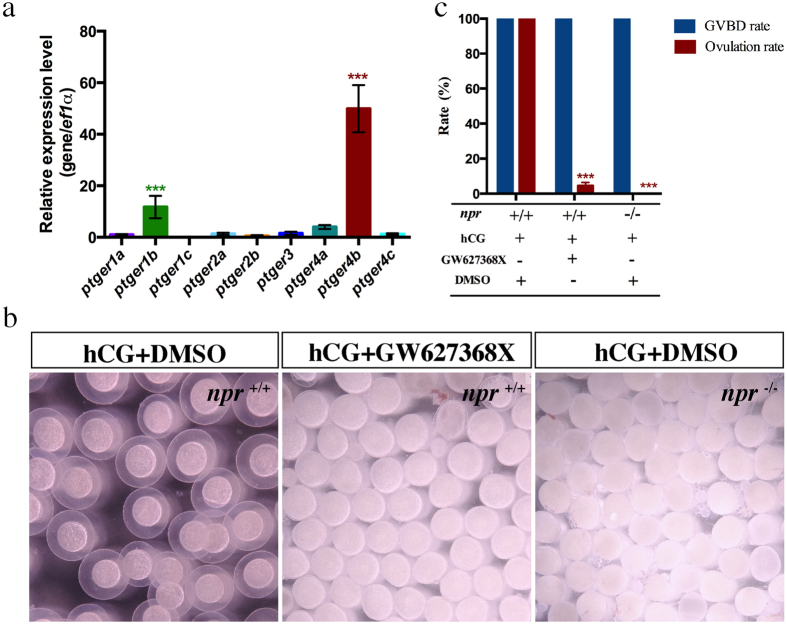
*In vivo* action of Ptger4 antagonist (GW627368X) on hCG induced oocyte maturation and ovulation. (**a**) Relative expression of *ptger* genes in FG stage follicles. (**b**) Morphology of follicles from *npr*^+/+^ and *npr*^−/−^ fish treated with hCG(10 IU/μl) or hCG + GW627368X(10 μM) for 4 hours. (**c**) Oocyte maturation and ovulation rates in *npr*^+/+^ and *npr*^−/−^ fish treated with hCG or hCG + GW627368X for 4 hours. Each value represents the mean value ± SEM of triplicate assays from 3 independent experiments. (***P < 0.001 vs control).

To investigate whether the *npr* downstream genes are functionally involved in ovulation, we investigated the roles of *ptger4b* which is the most up-regulated gene by hCG (Fig. 5). A search of PGE_2_ receptor genes reveals that the zebrafish genome contains nine different genes encoding for the PGE_2_ receptor. Of the nine *ptger* genes, the *ptger4b* was most predominant expressed (Fig. 6a). An antagonist of *ptger4b* (GW627368X) was employed to block *ptger4b* action. Interestingly, co-injection of GW627368X with hCG could effectively block the hCG-induced oocyte ovulation but not oocyte maturation (Fig. 6b), demonstrating that activation of *ptger4b* by LH signaling is required for successful ovulation. This effective blockade of ovulation by the *ptger4b* antagonist was similar to the ovulation defects observed in the *npr* null zebrafish after hCG injection (Fig. 6c), suggesting that both *npr and ptger4b* are downstream factors of LH signaling required for ovulation in zebrafish.

## Discussion

It is well established that LH signaling plays an important role in female ovulation in vertebrates including fish^2^,^5^. However, the downstream factors mediating LH action remain largely unknown. In this study, we have provided *in vivo* evidence that *npr* is an essential mediator of LH signaling on ovulation possibly through *ptger4b* in zebrafish.

Progesterone has been identified as an essential factor in female reproduction across vertebrates^28^,^29^. However, the receptor(s) that mediates the action of progesterone has been a subject of much debate for a long time^30^,^31^,^32^,^33^,^34^. Both the nuclear progestin receptor (*npr*)^35^ and a pair of membrane progestin receptor (*mprα* and *mprβ*)^36^ have been identified in zebrafish. All of them are expressed in zebrafish ovaries^34^,^35^,^36^,^37^,^38^. In order to provide clues to the functional roles of these receptors, we examined the expression levels of these receptors during folliculogenesis. The expression level of *mprβ* is increased from PG to PV stage follicles, similar to a report using nonquantitative gel electrophoresis^37^. Interestingly, the *npr* mRNA level is much higher than that of the *mprα* and *mprβ* levels during folliculogenesis, reaching the highest level at FG stage just prior to final oocyte maturation and ovulation. These data strongly argue for a pivotal role of *npr* on the regulation of final oocyte maturation or ovulation in zebrafish.

The ovarian expression patterns of *npr* and *lhr* are similar in zebrafish^39^, thus prompting us to investigate whether expression of the progesterone receptors is regulated by LH signaling. We found that *npr* is responsive to hCG, a human gonadotropin that has been widely used in fish to mimic the endogenous gonadotropin(s)^40^. Moreover, the regulation of *npr* by LH signaling is further substantiated by zebrafish mutant lines lacking *lhβ* or *lhr*^2^. Among all these receptors expressed in the ovary, only *npr* expression is dramatically decreased in the *lhβ* or *lhr* mutant fish. Using primary cultured zebrafish follicular cells, we have further demonstrated that *npr* is indeed upregulated by hCG, possibly through the cAMP-PKA pathway. The regulation of *npr* expression by LH signaling has also been observed in rat, mouse and medaka^41^,^42^,^43^, suggesting that this phenomenon is conserved in many species.

Although the functional roles of *npr* in mediating the action of LH signaling have been investigate in mammal^15^,^16^, studies in non-mammalian vertebrates lag behind. Recent studies suggested that *npr* may also regulate the ovulatory process in fish^43^,^44^, but robast genetic evidence is lacking. Using our optimized TALENs system, we have generated the *npr* fragment deletion mutant zebrafish line. We found that the *npr* null male zebrafish are fully fertile while the *npr* mutant female zebrafish are infertile. Folliculogenesis is normal in the *npr* mutant but ovulation is disrupted. Histological analysis reveals that mature oocytes are released from the surrounding follicle wall into the ovarian cavity in the ovaries of the wide-type but not the mutant. Similar phenotypes were also observed in a recent study^45^. These results clearly indicate that *npr* is indispensible for ovulation in zebrafish. In mice, targeted deletion of the progesterone receptor gene also leads to profound and complete anovulation, with the oocytes retained in the unruptured follicles even hyper-stimulated with gonadotropins^12^,^46^. It appears that *npr* function is well conserved from fish to mammals.

In order to understand the molecular mechanism of the action of LH on ovulation, we have established an *in vivo* ovulation model in zebrafish by hCG injection. A number of putative ovulation-related genes described in fish and mammals including the prostaglandin biosynthesis genes, various proteases, protease inhibitors and steroid biosynthesis genes were chosen and their expression profiles in *npr*^+/+^ and *npr*^−/−^ fish were analyzed after hCG injection. Down-regulation of *cpla2*, *ptgs2b*, *mmp15*, *3β-hsd* and *cyp19a1a* expression and up-regulation of *mmp9* expression were observed in the *npr* mutant, suggesting that these ovulation-related genes were regulated by *npr* signaling in zebrafish. Several genes including *cpla2*, *ptgs2a* and *timp2b* were up-regulated in both *npr*^+/+^ and *npr*^−/−^ fish after hCG injection, suggesting that these genes may also be involved in ovulation but in an *npr*-independent manner. More interestingly, we have identified two genes, namely *ptger4b* and *adamts1*, which were induced by LH signaling in an *npr*-dependent manner. The most responsive one is *ptger4b*, which is a receptor for PGE_2_. Prostaglandin (PG) has long been regarded as a critical regulator of ovulation in mammalian species^47^,^48^,^49^. The role of PG in mediating ovulation has also been studied in several fish species^26^,^50^,^51^,^52^,^53^,^54^,^55^,^56^. Interestingly, we found that the *pgter4* antagonist (GW627368X) could block hCG induced ovulation. These findings suggest that *ptger4b* is a downstream factor of *npr* in inducing ovulation upon stimulation by the endogenous LH surge.

In summary, the present study provides robust *in vivo* evidence indicating that *npr* mediates the action of LH signaling on ovulation possibly through *ptger4b* in zebrafish. Information obtained from the present study helps to elucidate the biochemical pathways that link the initial stimulation by LH to the actualization of ovulation.

## Methods

### Zebrafish husbandry

AB strain zebrafish were reared in the laboratory of the Chinese University of Hong Kong and Sun Yat-Sen University, following the protocols described in Westerfield^57^. Briefly, fish were maintained in flow-through aquaria under an artificial photoperiod of 14 hours light (9:00–23:00): 10 hours dark (23:00–9:00) at 28 ± 1 °C. The larval and adult zebrafish were fed with brine shrimp (hatched from eggs in 20 mL in 4L salt water) daily. All animal experiments were conducted in accordance with the guidelines and approval of the respective Animal Research and Ethics Committees of the Chinese University of Hong Kong and Sun Yat-Sen University.

### Isolation of ovarian follicles

The staging system we adopted in this study was based on recent studies^8^,^58^. The ovaries were carefully dissected out from 15–20 female zebrafish after anesthetization and decapitation, and placed in a 100-mm culture dish containing 60% Leibovitz L-15 medium. Follicles of different stages were manually isolated and grouped into the following stages: primary growth (PG) (stage I; below 0.1 mm in diameter), previtellogenic (PV) (stage II; ~0.30 mm in diameter), early vitellogenic(EV) (stage III; ~0.40 mm in diameter), midvitellogenic (MV)(stage III; ~0.50 mm in diameter), and full grown but immature (FG) (stage III;~0.65 mm in diameter). The isolation process normally lasted for 4–6 hours at room temperature before incubation and drug treatment at 28 °C for different periods of time.

### Primary culture of ovarian follicular cells

Primary culture of zebrafish ovarian follicular cells was performed according to an established protocol^59^. Briefly, follicles of vitellogenic stage from 15 to 25 females were carefully selected and washed with M199. The follicles were then cultured in a 25 cm^2^ flask for 6 days in M199 medium plus 10% fetal bovine serum under the condition of 28 °C and 5% CO_2_. The medium was changed on the third day. After 6 days, the cells were subcultured in 24-well plates at a density of 100 000 cells per well for 24 hours before hormone or drug treatment.

### RNA isolation and RT-PCR

Total RNA was extracted from the ovarian follicle samples, cultured follicular cells or ovary of zebrafish using TRIzol reagent (Invitrogen). The cDNA was produced from 1μg total RNA using Rever Tra Ace α-first strand cDNA Synthesis Kit (TOYOBO) and used as template. The specific primers used in this study are listed in Supplemental Table 1. The transcription levels of the target genes were measured using the SYBR Green PCR Master Mix Kit^60^ carried out on an ABI Real-Time PCR Fast System^60^. Quantitative RT-PCR conditions were as follows: denaturation at 95 °C for 1 minute, followed by 40 cycles of 95 °C for 15 seconds, 58 °C for 15 seconds, 72 °C for 20 seconds, and then 84 °C for 10 seconds (fluorescent data collection). All mRNA quantification data were normalized to *ef1α* and expressed as the fold differences of the target gene expression relative to the control.

### Establishment of an *npr* gene fragment disruption zebrafish line by TALEN

The TALENs were assembled using the golden gate method as described previously^61^,^62^. The two TALEN somatic expression backbones pCS2-TALEN-ELD and pCS2-TALEN-KKR were developed by our group^62^. For detailed protocol of the TALEN preparation, see ref. 61. TALEN mRNAs (200–500 pg) were microinjected into one-cell stage zebrafish embryos. Two days after injection, genomic DNA was isolated from 8–10 pooled larvae. The target genomic regions were amplified by PCR. To obtain germline mutations, the TALEN-injected embryos were raised to adulthood and the P0 founders were outcrossed with wild-type fish. The F1 progeny were genotyped by sequencing. To obtain homozygous mutants, heterozygous mutant of the same mutation were obtained and crossed. The primers used in this study are listed in Supplemental Table 1.

### PCR genotyping

To assess TALEN induced mutation of the *npr* target region and to determine the mutation rate, PCR genotyping was performed. Genomic DNA was extracted by the phenol-chloroform method from the tail fin of F2 fish. Genomic PCR was performed two reverse primers specific for the *npr*^+*/*+^(439 bp) or *npr*^−/−^ (355 bp) and a common forward primer as listed in Supplementary Table 1. The PCR products were electrophoresed on 1.5% agarose gels to resolve and identify the mutations.

### Morphological and histological analyses of the zebrafish mutant line

Morphological and histological analyses were performed as described^63^. Briefly, gross morphology of the adult fish was analyzed at 75 days post fertilization (dpf). Fish were euthanized using MS-222 and images were taken using a digital camera. Body length and body weight were measured. Then the ovary was isolated from the body cavity for histological examination after noting the gonad weight. The gonad-somatic index (GSI) was calculated as (gonad weight/body weight) × 100%. For gonad histology, the ovarian samples were fixed in Bouin’s solution overnight at room temperature. The samples were dehydrated through a graded series of ethanol and embedded in paraffin wax. The samples were serially cut into 7 μm sections on a Leica microtome. After rehydration, the sections were stained with hematoxylin and eosin and mounted with Canada balsam (Sigma-Aldrich) for microscopic examination.

### Fertility assessment

Fertility assessment was performed as described^63^. The fertility of *npr*^−/−^ female fish was assessed by natural mating with wild-type males in a spawning tray. One hour after light on in the morning, the spawned eggs were collected. Individuals that failed to spawn or produce fertilized embryos after at least 10 trials were considered infertile^5^.

### Maturation assay

Maturation assay was performed as described^8^. Female zebrafish were sacrificed and ovaries excised as described above. FG stage follicles from the mutant line and wild-type fish were collected and incubated (30–40 follicles/well) in 24-well culture plates at 28 °C. After treatment with 17α, 20β-DHP (Sigma-Aldrich) or recombinant zebrafish IGF3^8^, follicles that underwent GVBD were identified by their ooplasmic clearing (due to proteolytic cleavage of vitellogenin). Each group had 4 replicate wells, and each experiment was repeated 3 times.

### Induction of ovulation *in vivo*

hCG (Sigma-Aldrich) was dissolved in sterile distilled saline solution (NaCl 0.7%) at a concentration of 10 IU/μl. GW627368X (Cayman) was dissolved in DMSO at a concentration of 10 mM and then dissolved in sterile distilled saline solution (NaCl 0.7%) at a concentration of 10 μM. Before the administration of these agents, adult female fish were anesthetized. Fish were injected intraperitoneally with a volume of 5 μl/g body weight. After injection, the fish were placed individually in the tank.

### Statistical analyses

All data were expressed as mean values ± SEM. P < 0.05 was considered statistically significant using one-way ANOVA, followed by Fisher’s least significant difference test using the GraphPad Software. Statistical comparisons of the expression levels between wild-type and mutant fish were conducted using an unpaired 2-tailed Student’s t test.

## Additional Information

**How to cite this article**: Tang, H. *et al*. Gene knockout of nuclear progesterone receptor provides insights into the regulation of ovulation by LH signaling in zebrafish. *Sci. Rep.* **6**, 28545; doi: 10.1038/srep28545 (2016).

## Supplementary Material

Supplementary Information
