# Supplementary material for: Gene knockout of nuclear progesterone receptor provides insights into the regulation of ovulation by LH signaling in zebrafish
Source: Sci Rep. 2016 Jun 23;6:28545. doi: 10.1038/srep28545 (PMC4917859; doi:10.1038/srep28545)
Supplement: Supplementary Information [file srep28545-s1.doc]

**Supplementary Information**

**Gene knockout of nuclear progesterone receptor provides insights into the regulation of ovulation by LH signaling in zebrafish**

Haipei Tang^1, 2^, Yun Liu^1, 2, 3^, Jianzhen Li^2, 3^, Yike Yin^1^, Gaofei Li^1^, Yu Chen^1^, Shuisheng Li^1^, Yong Zhang^1, 2^, Haoran Lin^1^, Xiaochun Liu^1*^, Christopher H.K. Cheng^2, 3*^

^1^State Key Laboratory of Biocontrol, Institute of Aquatic Economic Animals and Guangdong Province Key Laboratory for Aquatic Economic Animals, School of Life Sciences, Sun Yat-Sen University, Guangzhou 510275, China.

^2^School of Biomedical Sciences, The Chinese University of Hong Kong, Hong Kong, China.

^3^School of Biomedical Sciences Core Laboratory, The Chinese University of Hong Kong Shenzhen Research Institute, Shenzhen 518057, China.

*Corresponding authors:

Christopher H.K. Cheng, Rm 604A, 6/F., Lo Kwee Seong Integrated Biomedical Sciences Building, The Chinese University of Hong Kong, Shatin, N.T., Hong Kong, China. Tel: +852-39436801; E-mail: [chkcheng@cuhk.edu.hk](mailto:chkcheng@cuhk.edu.hk)

Xiaochun Liu, Institute of Aquatic Economic Animals, School of Life Sciences, Sun Yat-Sen University, Guangzhou 510275, China. Tel: +86-20-84112511; Fax: +86-20-84113717; E-mail: [lsslxc@mail.sysu.edu.cn](mailto:lsslxc@mail.sysu.edu.cn)

Supplemental Table 1. Primers used in the present study.

| **Primer name** | **Primer sequence (5′-3′)** | **Purpose** |
| --- | --- | --- |
| *npr* F1  *npr* R1 | CTTACACTGAACACATAGACGAC  GACTGATACCAGAACGGAGAGTC | Amplification and sequencing  of the *npr* locus |
| *npr* R2 | GTCCGTGTGGACTGGCATTGAGC |  |
| *npr* F  *npr* R | ACAGACAGCATACACCGC  TCCACAGGTCAGAACTCC | Real-time PCR of *npr* |
| *mprα F*  *mprα R* | CGCTCAAGTGCGAACTTTTT  CGTACTTGCCATAGCAGCAG | Real-time PCR of *mprα* |
| *mprβ F*  *mprβ R* | ACGTCAAGCCACAGTACACG  TCCTGATGCACTGGACGATA | Real-time PCR of *mprβ* |
| *cpla2 F* | TGCTCTTGGAAGTTTGCGC | Real-time PCR of *cpla2* |
| *cpla2 R* | TCTGCGTGTCTGCATGAACAG |  |
| *ptgs1* F | GAGGCAAGACAGGCTTCGAT | Real-time PCR of *ptgs1* |
| *ptgs1* R | ACATACGGGCACCATTTCGT |  |
| *ptgs2a* F | TTATGGAGAGACGCTGGAGGT | Real-time PCR of *ptgs2a* |
| *ptgs2a* R | GATGAGACGAGTGGTTTGGAA |  |
| *ptgs2b* F | GGCTCATCCTTATTGGTGAGACTAT | Real-time PCR of *ptgs2b* |
| *ptgs2b* R | TCGGGATCAAACTTGAGCTTAAAATA |  |
| *ptger1a* F | TCTCGCTTTCTGTCATCTGG | Real-time PCR of *ptger1a* |
| *ptger1a* R | TGCCATATCTGCCTTCTCAG |  |
| *ptger1b* F | ACATCTTGCATCTGCTGGAG | Real-time PCR of *ptger1b* |
| *ptger1b* R | CCGGTAGGACATTTCCAGAT |  |
| *ptger1c* F | TCTGCTGGAGTCCCTTTCTT | Real-time PCR of *ptger1c* |
| *ptger1c* R | AACGCAGAGCCAAGAGAAAT |  |
| *ptger2a* F | GCGTATTTGACCAACACCAC | Real-time PCR of *ptger2a* |
| *ptger2a* R | TCGCCAGGAGGATAGAAAGT |  |
| **Primer name** | **Primer sequence (5′-3′)** | **Purpose** |
| *ptger2b* F  *ptger2b* R | CGCATGTATAGGAGGCTGAA  TGACGATCTCGGTGGTAGAG | Real-time PCR of *ptger2b* |
| *ptger3* F | TCATCAAAGCAGTGGGAGAG | Real-time PCR of *ptger3* |
| *ptger3* R | GGTGACAGAGATCCAGCAGA |  |
| *ptger4a* F  *ptger4a* R | TCTCTGCTCATCCTGGTCAC  CTGGTTGAGAAACACCTGGA | Real-time PCR of *ptger4a* |
| *ptger4b* F  *ptger4b* R | GTGCCAGTATTCCGGGTTCA  AGAAGAGCGCATTGGACACA | Real-time PCR of *ptger4b* |
| *ptger4c* F | ATGCTCCATCCCTTTAGTGG | Real-time PCR of *ptger4c* |
| *ptger4c* R | AAGGAGGCGAATCGTATAGC |  |
| *mmp2* F | TCAGGGTCGAGATGATGGGT | Real-time PCR of *mmp2* |
| *mmp2* R | TGTGGTGCAGGAGTCGTAAC |  |
| *mmp9* F | GCTGCTCATGAGTTTGGACA | Real-time PCR of *mmp9* |
| *mmp9* R | AGGGCCAGTTCTAGGTCCAT |  |
| *mmp14* F | CTCTCCTAAATCTATCAACTCCGC | Real-time PCR of *mmp14a* |
| *mmp14* R | CTGCTTCATCAAAATGTGTGTCTC |  |
| *mmp15* F | AGGTGTGTTCGTGTGTCGTT | Real-time PCR of *mmp15a* |
| *mmp15* R | AGCCAAGACTCCGCATTGAA |  |
| *timp2b* F | CGTGAAGATAGTGTCAATCTCTC Real-time PCR of *timp2b* | |
| *timp2b* R | TCACCGGCAATGACGCTTATG |  |
| *3β-hsd* F  *3β-hsd* R | GGAGAGCTTGTAAAGGAGCAG  TCTCAAGCAGCAGCTTTGTT | Real-time PCR of *3β-hsd* |
| *17β-hsd3* F  *17β-hsd3* R | AAATTCTGGCGATTCATTCC  ACACCCATTGGTGGAAACTT | Real-time PCR of *17β-hsd3* |
| *cyp19a1a* F  *cyp19a1a* R | GTTGTCTCCTACTGTCGGTTCAT  CAGACCCAGTTTACTTCCAAAGC | Real-time PCR of *cyp19a1a* |
| **Primer name** | **Primer sequence (5′-3′)** | **Purpose** |
| *adamts1* F  *adamts1* R | GTTTGGCACACAATGACCTC  CAGCCTTCACACACTGTCCT | Real-time PCR of *adamts1* |
| *ctsl* F  *ctsl* R | GGCAGATCAGGGTTTGAAAT  GTCACATAGCCCTTGTCCCT | Real-time PCR of *ctsl* |
| *ef1α* F  *ef1α* R | GGCTGACTGTGCTGTGCTGATTG  CTTGTCGGTGGGACGGCTAGG | Real-time PCR of *ef1α* |
